# The "Cut It Out" Technique Approach in Open Globe Injury Management Caused by a Penetrating Fishhook Trauma

**DOI:** 10.7759/cureus.77986

**Published:** 2025-01-25

**Authors:** Alberta Y Tansil, Vera Sumual, Yuliana Hartono, Rillya Manoppo, Freili F Akay

**Affiliations:** 1 Department of Ophthalmology, Faculty of Medicine, Sam Ratulangi University, Manado, IDN

**Keywords:** cut-it-out technique, ocular fish hook injury, ocular trauma, open globe trauma, trauma pediatric

## Abstract

This case report explores fishhook-related ocular injuries, which vary in severity and may involve different ocular structures. It focuses on the "cut-it-out" technique for removing an embedded fishhook and highlights its effectiveness. Additionally, it emphasizes the necessity of immediate first aid and infection control in managing these complex ophthalmic cases.

A nine-year-old male patient arrived at our clinic six days post-injury. The ocular examination revealed a corneal laceration, edema, folds in Descemet's membrane, hypopyon, and a retained lens fragment. Visual acuity was significantly impaired. Emergency surgery was conducted under general anesthesia, encompassing wound debridement, paracentesis, extraction of the fishhook, and wound closure. Postoperatively, the patient had a marked enhancement in visual acuity.

Fishhook injuries pose a significant risk due to their barbed structure and potential for bacterial contamination. Timely diagnosis and surgical intervention are essential to minimize tissue damage and prevent complications such as infection, cataract formation, vitreous hemorrhage, and retinal detachment. Prophylactic antibiotics and meticulous postoperative care are crucial. Protective eyewear during fishing activities is strongly recommended to reduce the incidence of such injuries, and public awareness campaigns should be promoted.

Fishhook ocular injuries can have devastating consequences for vision, potentially leading to blindness. Prompt diagnosis, immediate surgical intervention, and appropriate postoperative care are crucial for optimal patient outcomes. While the "cut-it-out" technique proved effective, the optimal management approach may vary depending on the specific injury characteristics. Further research is needed to refine our understanding of these injuries and establish evidence-based treatment guidelines.

## Introduction

Ocular injuries are a leading cause of preventable vision loss, especially among children, with significant psychological, social, and economic impacts [1,2]. Penetrating eye trauma from fishhooks is particularly alarming due to its potential to cause severe and permanent vision impairment if not treated properly. On a global scale, an estimated 1.6 million people are completely blind, and an additional 2.3 million experience significant visual impairment as a result of ocular trauma. Children between the ages of nine and 11 are particularly susceptible, with males exhibiting a higher incidence rate [2].

Among various types of ocular trauma, fishhook injuries stand out as both unique and incredibly challenging [2]. The barbed structure of fishhooks complicates their removal and heightens the risk of serious eye complications, such as corneal lacerations, hyphema, cataracts, vitreous hemorrhage, and retinal detachment [1,2]. Timely and appropriate treatment is essential to improve visual outcomes and reduce the risk of long-term vision damage.

This case report details a young patient who sustained a fishhook-related ocular injury and was successfully treated using a "cut-it-out" technique. This approach emphasizes the importance of prompt, specialized care and demonstrates that positive outcomes are possible even in severe cases. The report aims to underscore this technique's effectiveness and the critical role of immediate first aid and infection prevention in managing fishhook-related eye injuries, a challenging scenario for ophthalmologists.

## Case presentation

**Figure 1:**
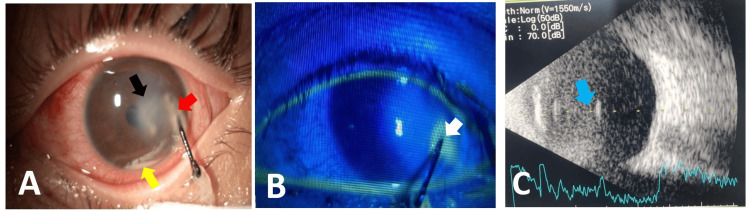
Pre-operative and Ultrasonography. A. A fishhook penetrates the anterior chamber of the eye, having an entry wound at four o'clock (red arrow). Corneal edema, Descemet folds, and a detached lens (black arrow) are visible. Hypopyon is seen (yellow arrow). B. Fluorescein staining did not reveal corneal epithelial defects or aqueous humor leakage. C. Conversely, ultrasonography detected the presence of a point-like lesion, suspecting endophthalmitis (blue arrow)

**Figure 2:**
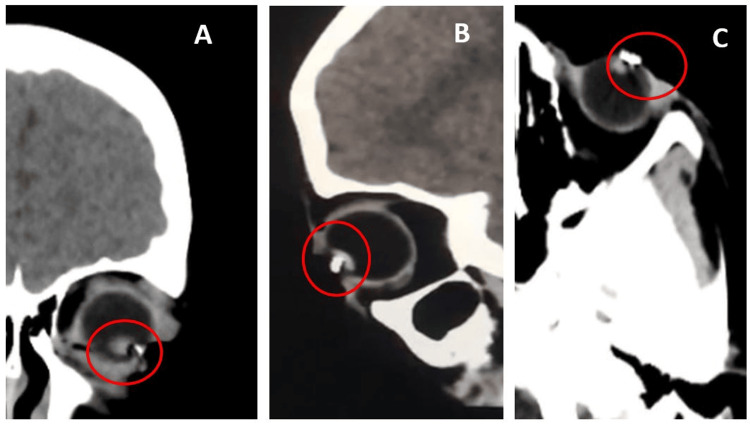
Head CT Scan Series A. Coronal view. B. Sagittal view. C. Axial view showing a metallic foreign matter within the left globe extending to the anterior chamber (red circle).

A nine-year-old boy presented six days after a fishhook became embedded in his left eye. The patient's affected eye exhibited a best-corrected visual acuity of 2.0 LogMAR, and intraocular pressure was deemed moderate upon digital palpation. Slit-lamp examination revealed a 3 mm corneal laceration at the four o'clock position, along with corneal edema and Descemet's folds. Hypopyon and lens fragments were seen in the anterior chamber (Figure 1A). Corneal fluorescein staining showed no epithelial defects or leakage of aqueous humor (Figure 1B). Ultrasound biomicroscopy confirmed the presence of a point-like lesion, suspecting endophthalmitis (Figure 1C). Cranial computed tomography (CT) imaging identified a metallic foreign body lodged in the left eye, extending into the anterior chamber (Figure 2).

**Figure 3:**
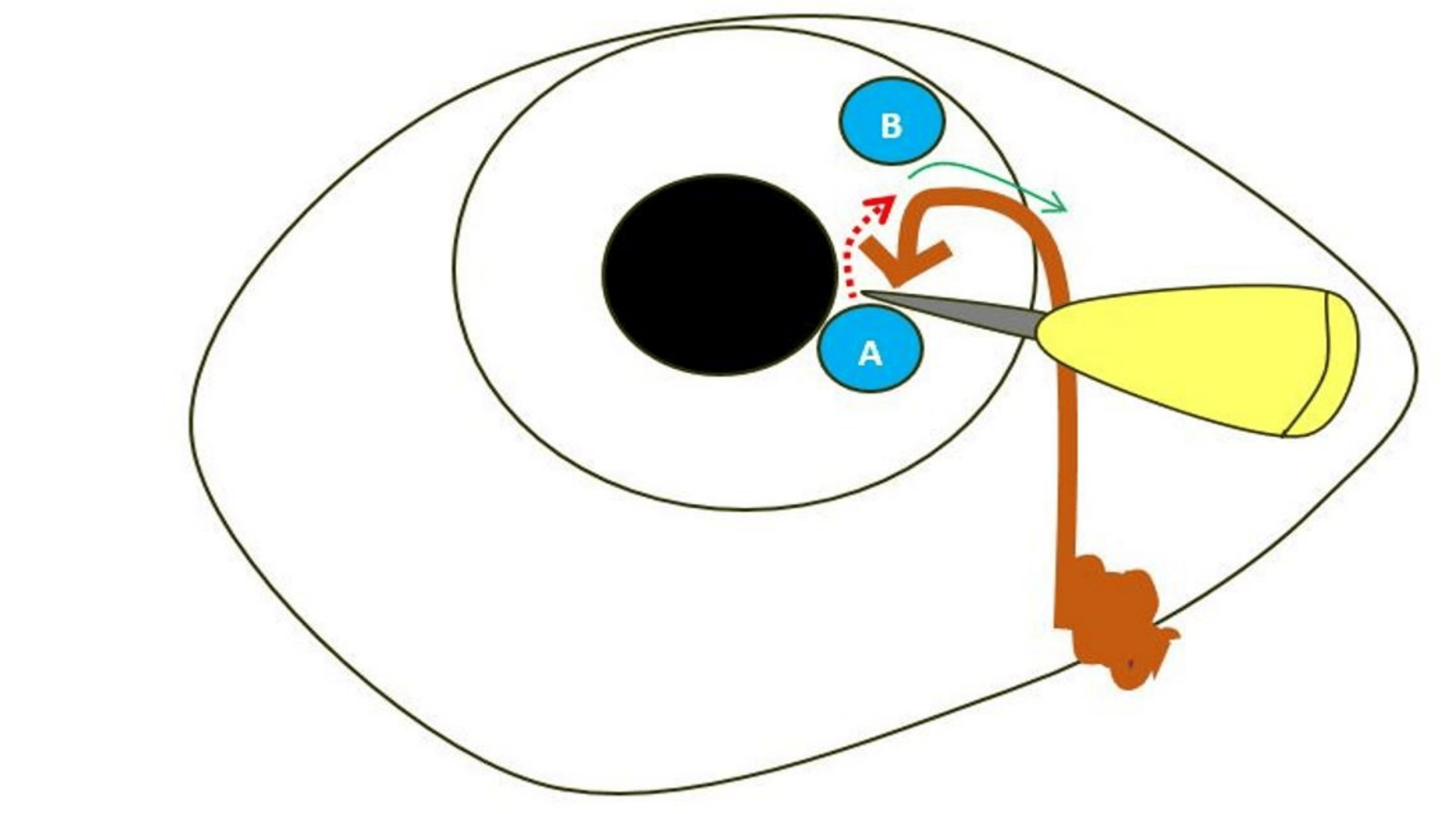
Illustration of the steps for fishhook extraction techniques A. A 15-number blade was used to enlarge the entry wound (red arrow). B. The fishhook was then carefully removed through the same wound using a gentle, angled maneuver (green arrow).

Under general anesthesia, emergency surgery was performed to manage the fishhook injury. The wound was meticulously irrigated with balanced saline and antibiotic solutions. Two small incisions were created to facilitate pus drainage, and a viscoelastic agent was applied to maintain the structural integrity of the eye. The fishhook, which had penetrated both the cornea and iris, was carefully extracted following controlled enlargement of the incision (Figure 3). The wound was then meticulously sutured to restore the anatomical integrity of the eye. Subsequently, lens extraction was performed to address traumatic cataract formation. To minimize the risk of infection, an intravitreal antibiotic injection was administered.

**Figure 4:**
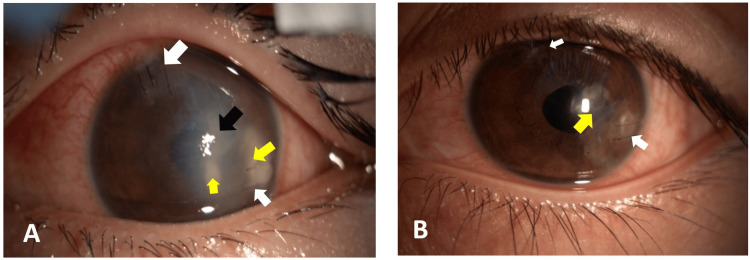
Post-operative A. Day 1: The anterior segment of the left eye showed corneal edema, Descemet’s folds (black arrow), a cicatricial cornea (yellow arrow), a sutured cornea (white arrow), and aphakia. B. Day 7: The anterior segment of the left eye revealed a cicatricial cornea (yellow arrow), a sutured cornea (white arrow), and aphakia.

Postoperative management included oral and topical antibiotics, topical corticosteroids, and topical cycloplegic (atropine). The patient was discharged the following day. Subsequent follow-up examinations at one and four weeks revealed no complications. Visual acuity in the affected eye showed significant improvement, rising from 2.0 LogMar to 0.2 LogMar within one week, with stable results maintained over the subsequent month. No recurrence of hypopyon or corneal wound complications was noted (Figure 4). Intraocular lens (IOL) implantation is planned for a future stage.

## Discussion

Fishhook-related ocular injuries, as demonstrated in this case, present a unique and significant clinical challenge that requires meticulous diagnosis and management. The fishhook in this instance penetrated the cornea and extended into intraocular structures, leading to complications such as corneal edema, hypopyon, and traumatic cataract. Early surgical intervention and precise techniques are essential to preserving visual function and minimizing long-term complications.

**Figure 5:**
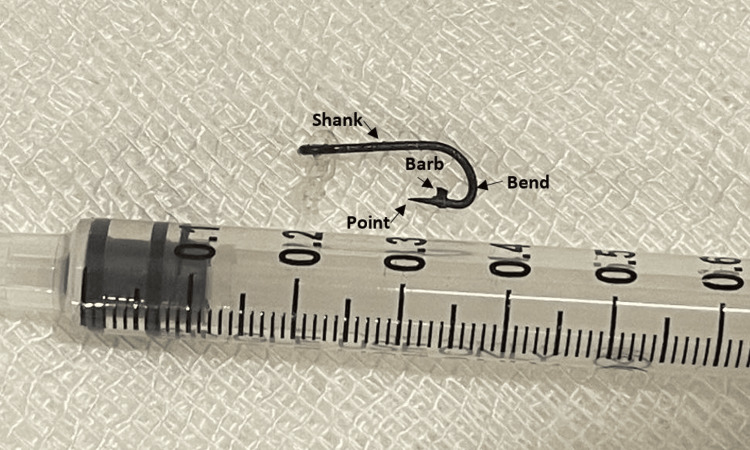
The fishhook on extraction

The diverse types and sizes of fishhooks, each with distinct anatomical features, necessitate a thorough understanding of their structure for optimal management. A fishhook typically consists of five key components: the eye, shank, bend, barb, and tip. Each of these elements can complicate removal and increase the risk of ocular damage (Figure 5) [3,4].

Detailed examination of the fishhook’s configuration - including the number and presence of barbs and its overall size and shape - is crucial for determining the most suitable extraction technique. Barbed fishhooks, in particular, pose a heightened risk of severe ocular complications, such as corneal lacerations, intraocular foreign bodies, and vision-threatening infections. Insights from the angler regarding the type of hook used can provide valuable guidance for selecting the appropriate management strategy [5].

Fishhook injuries often result in a broad spectrum of ocular complications due to their intricate design and unpredictable behavior. Corneal penetration, as seen in this case, is a common finding and can lead to significant corneal scarring, which may adversely impact visual acuity. While long-term visual prognosis is typically favorable unless scarring is extensive, the risk of more serious complications remains [2,6].

The fishhook’s penetration into the lens capsule caused fragmentation and initiated chronic intraocular inflammation. If untreated, these sequelae could progress to secondary glaucoma and irreversible visual impairment [7,8]. Additionally, injuries involving the posterior segment of the eye may result in choroidal and vitreous hemorrhage, retinal tears, and, in severe cases, complete retinal detachment.

Open-globe injuries, particularly those involving contaminated foreign objects such as fishhooks, significantly elevate the risk of intraocular infection. Studies report that the incidence of endophthalmitis in such cases ranges from 4% to 13% without prophylactic treatment [4,6]. Timely and tailored surgical intervention is critical to mitigating these risks and preserving vision. Most fishhook injuries (approximately 70%) involve the anterior segment of the eye, particularly the cornea. Careful assessment of the fishhook's position, depth of penetration, and surrounding tissue damage is indispensable for determining the most appropriate extraction technique and preventing additional injury during removal.

The "cut-it-out" technique was utilized to safely remove the fishhook while minimizing further tissue trauma. This approach was deemed optimal due to the fishhook’s structural characteristics, particularly the presence of a barb, which would exacerbate tissue damage if removed via direct traction. Alternative methods, such as the retrograde technique (retracting the hook while twisting) and the needle-cover technique (using a needle to shield the barb before extraction), were considered less appropriate given the fishhook's penetration into both the cornea and iris. Furthermore, corneal edema and hypopyon impaired intraoperative visualization, reinforcing the need for a controlled and precise removal technique. Evidence from the literature supports the "cut-it-out" method as effective in managing complex fishhook injuries, particularly when delicate surrounding tissues are at risk of further damage [6,7].

Lens extraction was performed to address the traumatic cataract and mitigate the risk of secondary complications, such as chronic intraocular inflammation and secondary glaucoma. Literature supports the early removal of the damaged lens in cases of traumatic cataract to prevent recurrent inflammation and optimize visual outcomes [9].

Prophylactic antibiotic therapy was administered to prevent endophthalmitis, a recognized complication of open-globe injuries involving contaminated foreign objects. Despite the absence of overt clinical signs of infection at the time of presentation, intravitreal and systemic antibiotics were used as a precautionary measure. Fishhooks are often contaminated with environmental bacteria or skin flora, necessitating proactive infection prevention even in cases with minimal epithelial damage [5]. Close postoperative monitoring for signs of infection remains essential.

The patient demonstrated remarkable improvement in visual acuity, progressing from 2.0 LogMAR to 0.2 LogMAR within one week postoperatively, highlighting the success of the intervention. Plans for IOL implantation are underway, with the procedure scheduled once ocular stability is achieved. This is expected to further enhance visual outcomes. Although corneal scarring is a frequent sequela of fishhook injuries, the overall visual prognosis remains favorable, provided long-term complications such as glaucoma and chronic inflammation are effectively managed [3,6].

## Conclusions

Managing ocular fishhook injuries requires a comprehensive, multidisciplinary approach to achieve the best visual outcomes for patients. Prompt surgical intervention, using techniques like the "cut-it-out" method, can effectively remove deeply embedded hooks and minimize further tissue damage. Careful postoperative monitoring and proactive management of complications, such as endophthalmitis, traumatic cataract, and retinal detachment, are crucial. While the final visual prognosis may vary, appropriate and timely treatment can help many patients achieve favorable outcomes despite the initial severity of the injury.
